# Pacemaker lead extraction saved a severe lead-induced tricuspid regurgitation: a case report

**DOI:** 10.1093/ehjcr/ytae560

**Published:** 2024-10-18

**Authors:** YuLiang Chai, Qiang Liu, Zhiwen Chen, Wenjing Zhang, Yuanqing Liu

**Affiliations:** Department of Cardiology, Jiangxi Provincial People’s Hospital, The First Affiliated Hospital of Nanchang Medical College, No. 92, Aiguo Road, Nanchang City, Jiangxi Province 330006, China; Department of Cardiology, Jiangxi Provincial People’s Hospital, The First Affiliated Hospital of Nanchang Medical College, No. 92, Aiguo Road, Nanchang City, Jiangxi Province 330006, China; Department of Cardiology, Jiangxi Provincial People’s Hospital, The First Affiliated Hospital of Nanchang Medical College, No. 92, Aiguo Road, Nanchang City, Jiangxi Province 330006, China; Department of Cardiology, Luoyang Central Hospital Affiliated to Zhengzhou University, 288 Zhongzhou Middle Road, Xi Gong Qu, Luo Yang City, He Nan Province 471000, China; Department of Cardiology, Jiangxi Provincial People’s Hospital, The First Affiliated Hospital of Nanchang Medical College, No. 92, Aiguo Road, Nanchang City, Jiangxi Province 330006, China

**Keywords:** Tricuspid regurgitation, Heart failure, Case report, Pacemaker lead extraction, Left bundle branch area pacing

## Abstract

**Background:**

Pacemaker lead-induced tricuspid regurgitation is a common complication after cardiac implantable electronic device (CIED) implantation. Cardiac implantable electronic device lead removal is a challenge procedure.

**Case summary:**

A 72-year-old lady was admitted due to worsening heart failure. She had a history of permanent atrial fibrillation and had a permanent single-chamber pacemaker implanted 8 years ago due to complete heart block. Transthoracic echocardiography identified severe lead-related tricuspid regurgitation. The patient underwent successful lead extraction and received a new implantation of left bunch bundle area pacing. Transthoracic echocardiographic examination 2 days after the procedure showed a significant decrease of the tricuspid regurgitation. The patient also reported an improvement in heart failure symptoms.

**Discussion:**

Pacemaker lead-related tricuspid regurgitation introduces negative haemodynamic overload, carrying high risk for the development of heart failure and worse outcome. The present case shows a rapid relief of symptom and improvement of echocardiography findings, indicating the significance of mechanistic approach in the treatment of lead-related tricuspid mechanical interference.

**Learning points**

1. A manual pacemaker lead extraction can still be feasible in selected patients with lead-induced tricuspid regurgitation. Clinical evaluations are vital for the selection.
2. No sign of calcification in echocardiography and a passive lead tip may be good conditions preferred by a manual pacemaker lead extraction.
3. The lumen-less Medtronic 3830 lead interferes less tricuspid mobilities and may thereby cause less lead-related tricuspid regurgitation compared with the usual pacemaker leads.

## Introduction

With increasing pacemaker implantation, lead-related tricuspid regurgitation (TR) is not uncommon. It has been shown that it accounts for 25% of the TR and may increase mortality of patients.^1^ Guideline for the standard treatment of lead-related regurgitation is still under the development. We presented a case of manual lead extraction to treat a large lead-induced TR.

## Summary figure

**Figure:**
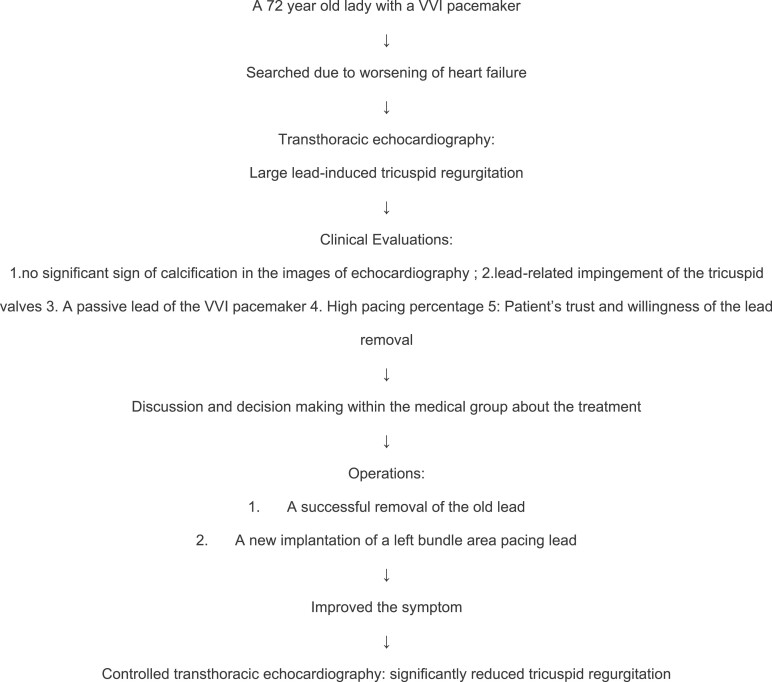


## Case presentation

A 72-year-old woman was admitted due to worsening heart failure. She had had symptoms of effort-induced chest pressure and breathlessness for 10 years but had experienced a worsening of the symptoms in the last 2 weeks. Her medical history included chronic anaemia, permanent atrial fibrillation, and a permanent single-chamber pacemaker (VVI), implanted 8 years ago due to complete atrioventricular block.

**Figure 1:**
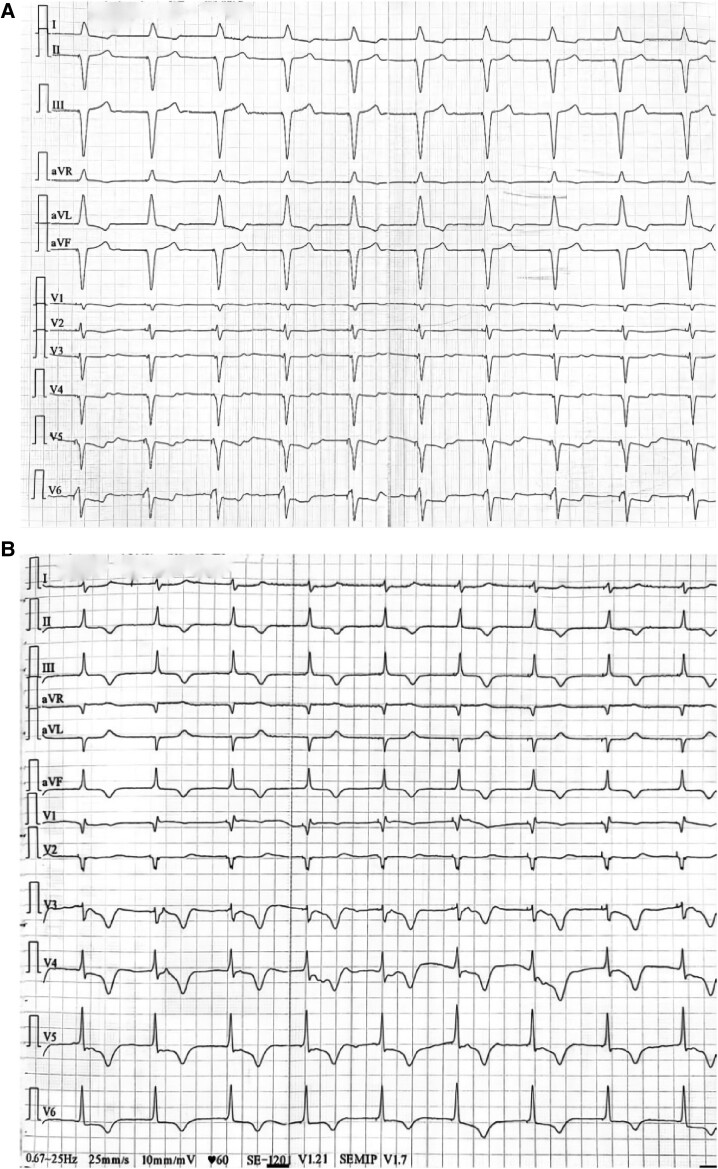
(*A*) Electrocardiogram showing chronic atrial fibrillation with ventricular pacing paced QRS duration 160 ms before lead extraction. (*B*) Electrocardiogram showing left bundle branch pacing with a narrowed QRS complex of about 110 ms and typical qR waves in V1 after the lead extraction.

At admission, she had normal vital signs: blood pressure 125/60 mmHg, pulse 60 b.p.m., respiratory rate 20 breaths/min, and temperature 36.3°C. The cardiac enzymes were negative. Her NT-proBNP levels were elevated 3253 pg/mL (normal range: 0–290.2 pg/mL); and her heart rate was 60 b.p.m. A 24 h electrocardiogram (ECG) monitoring showed 98% ventricular pacing. The ECG recorded at admission showed atrial fibrillation with ventricular pacing, paced QRS duration 160 ms (*Figure 1A*).

**Figure 2:**
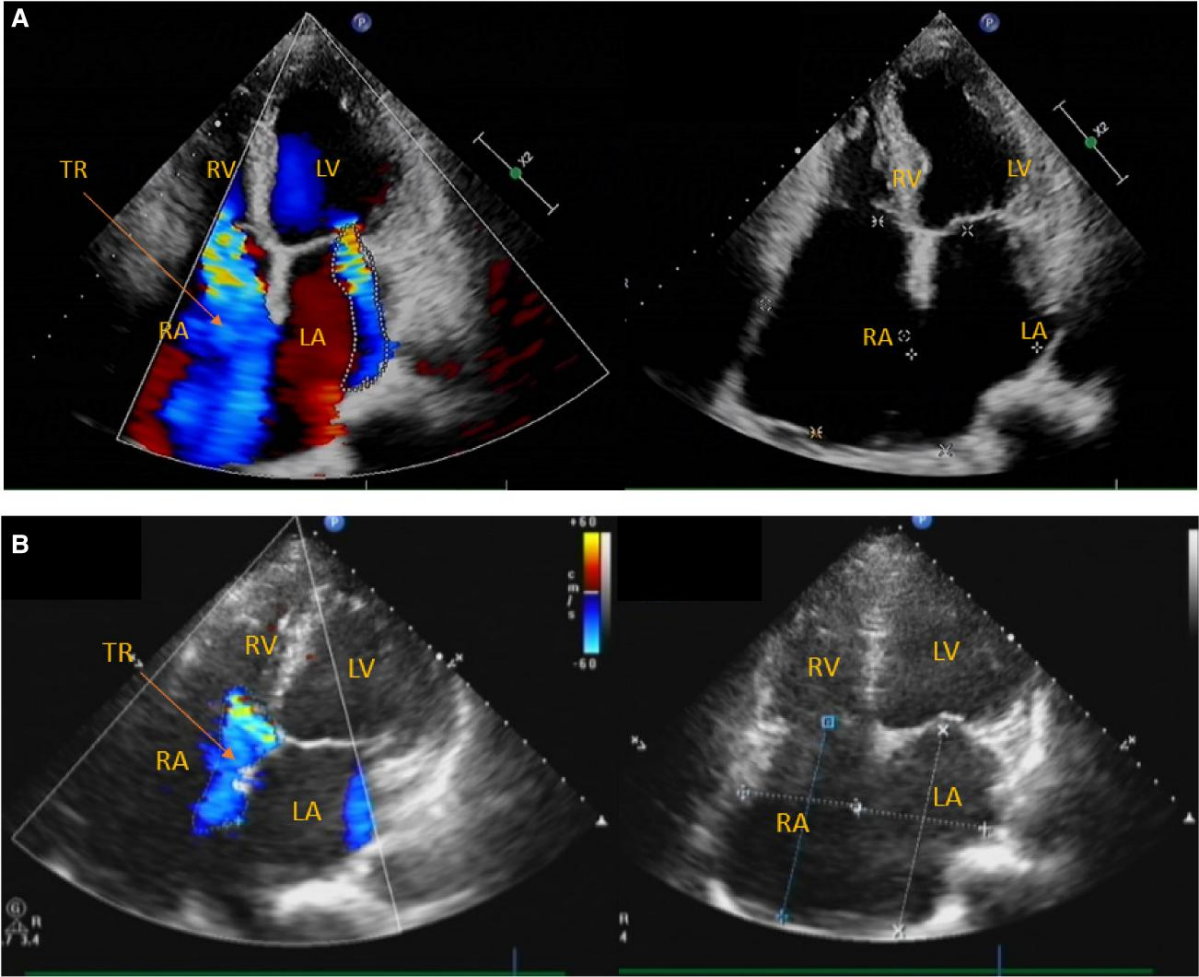
(*A*) Echocardiography showing large lead-related tricuspid regurgitation (pointed by the arrow) before lead extraction (ejection fraction: 50%, the end-diastolic dimension of the left ventricle: 53 mm, the area of tricuspid regurgitation: 20 cm^2^, Vmax of the tricuspid regurgitation: 3 m/s, pressure gradient across tricuspid valves: 35 mmHg; right atria size: 51.3 cm^2^, the left atria size: 24 cm^2^). (*B*) Echocardiography showing significantly reduced tricuspid regurgitation (like the arrow pointing) and decreased atrial size after the lead extraction (EF: 62%, LVED: 57 mm, the area of tricuspid regurgitation: 8.1 cm^2^, Vmax of the tricuspid regurgitation: 3 m/s, pressure gradient across tricuspid valves: 35 mmHg; the right atria size: 39 cm^2^, the left atria size: 22.8 cm^2^).

Transthoracic echocardiography (*Figure 2A*) showed an ejection fraction 50%, an end-diastolic dimension of the left ventricle 53 mm and an end-systolic dimension of the left ventricle 39 mm, and a large lead-related TR (according to the echocardiographic reports, it was shown a lead-related impingement of the tricuspid valves with a regurgitation jet area of 20 cm^2^); the right atria was significantly enlarged, measuring 51.3 cm^2^; the left atria area was 24 cm^2^. A medium mitral valve regurgitation was also observed. Non-significant sign of calcification in any of the heart valves was shown in the echocardiography. Moreover, her red blood cell count was 2.56 × 10^12^/L (normal range: 3.8–5.1 × 10^12^/L) and hemoglobin was 85 g/L (normal range: 115–150 g/L), which were stable as before.

A pacemaker control showed that the patient had high right ventricular pacing and needed an imminent change of her pacemaker battery. The thoracic surgeons were consulted. The patient had a strong wish to avoid surgery and preferred a lead extraction to any open thoracic operation. Considering the findings of severe lead-induced TR on the ultrasound examination, the heart team decided pacemaker extraction with the backup of thoracic surgery and thereafter a new implantation of left bundle branch area pacing (LBBAP) due to high right ventricular pacing as the best treatment approach. The patient had had a VVI system for 8 years and a sole ventricular lead with a passive tip. Moreover, the thoracic ultrasound did not show any sign of significant calcification of the heart valves. These conditions provided a relatively good opportunity for manual extraction of the ventricular lead.

The day after admission, the patient underwent a successful lead extraction. We applied a soft tract on the lead which was detached from the apex of the right ventricle without any use of laser aid. Thereafter, a new electrode aiming at LBBAP was implanted. Electrocardiogram after LBBAP implantation is shown in *Figure 1B* showing left bundle branch pacing with a narrowed QRS complex of about 110 ms and typical qR waves in V1.

An echocardiographic examination 2 days after the lead extraction showed significantly reduced TR (medium level according to the echocardiographic reports with regurgitation jet area of 8.1 cm^2^) and decreased atrial size (39 cm^2^ compared with 51.3 cm^2^ before the lead extraction) (*Figure 2B*). During the follow-up visit 8 weeks after the lead extraction, the patient experienced a great reduction in the symptoms (effort dyspnoea 1/5 Likert scale compared with 3–4/5 before lead extraction). NT-proBNP was also decreased to 738 pg/mL (compared with 3253 pg/mL at admission).

## Discussion

Lead-related TR is relatively common complications after device implantation, ranging affecting 10% to 39% of all cardiac implantable electronic device (CIED) implantation.^2^ The prevalence of moderate to severe TR has been reported to be as high as 5% 1 year after implantation.^3^ Echocardiography is a major tool to identify lead-related TR. Cardiac implantable electronic device–associated TR can be classified into lead-related mechanical interference on tricuspid valve coaptation, pacing-induced TR, and tricuspid valve dysfunction following lead extraction.^4^ In the present case, lead-related mechanical interference on tricuspid coaptation was, according to the ultrasound findings, defined as the primary cause for the hospitalization of the patient, though other factors such as persistent atrial fibrillation and enlarged atrial size may also partly contribute to the TR. Lin *et al.*^5^ have reported a 39% mechanical interference on leaflet mobility in 41 patients with severe TR caused by previously implanted permanent pacemaker or implantable cardioverter-defibrillator leads. Lead-induced TR in patients with left ventricular eject fraction < 40% has been shown to be associated with poor prognosis. The presence of moderate to severe TR is associated with increased all-cause mortality and heart failure, independent of left ventricular function, right ventricular pacing, and age.^6,7^ Pure medical therapy is not enough in the management of severe or symptomatic TR. Surgical interventions are recommended in current expert consensus statements to treat such severe symptomatic TR.^7^

Transvenous lead extraction is a part of an overall lead management strategy for lead-induced severe TR.^8^ However, lead extraction is a challenging procedure with high risk of complications. The major risk of the lead extraction procedure is tricuspid tissue avulsion during manual traction for lead removal, with a consequent worsening of the TR severity. Lead adherence initiates as soon as 4–5 days after implantation. Fibrosis and fibrotic binding is affecting the lead–valve-tissue complex progressing over time, with severe inflammation and calcification. This makes it challenging to conduct a transvenous lead extraction due to high risk for avulsion. In the present case, the tip of pacemaker lead seemed not involving extensive fibrosis or severe calcification. We tried with a simple manual traction approach by applying a mild manual extraction on the lead without using any laser or other lead extraction tools. According to the ELECTRa registry conducted by the European Heart Rhythm Association (EHRA), simple traction by applying mild pulling force without the use of specialized tools (other than a standard stylet) was used in 27% of patients in the registry.^9,10^ This manual approach may be mostly used for leads with a short dwell time (i.e. time since implant < 1–2 years). Besides, locking stylet, mechanical non-powered telescoping sheath and powered sheath can be used in the lead extraction. Laser sheaths, using laser energy delivered circumferentially along the tip of the sheath, are probably prevalent in centred tertiary hospitals for lead extraction. Previous studies have shown that laser-aided lead extraction is the predominant method of lead removal in the majority of patients.^11^ Snare, another tool for lead extraction, is most often deployed via a femoral approach and may consist of a single or double loop, which can be used for grasping free floating lead extremities or the lead body (double loop design). Moreover, in recent years, transcatheter therapies have emerged as a potential modality in the treatment of TR by using devices to perform transcatheter valve repair, orthotopic valve replacement, and the use of heterotopic devices with caval valve implants. Preliminary data of transcatheter therapies are promising. The first randomized trial of transcatheter valve repair by Sorajja *et al.*^12^ has shown that transcatheter tricuspid repair with the use of TriClip device resulted in significant improvement in TR severity and patient-reported quality of life. Similarly, Mao *et al.*^13^ have reported that transcatheter valve replacement with LuX-Valve has successfully reduced TR in six patients with lead-related TR. The number of patients included in those reported trials is limited, and data on long-term outcomes are missing. More randomized trials are needed to evaluate the safety and effect of transcatheter therapy in the treatment of lead-induced TR.

After lead extraction, a new electrode or device can be implanted depending on how much the patient needs ventricular pacing and the infectious status. If a patient undergoes lead extraction due to pocket infection or lead-related infection, an epicardial electrode or wireless Micra pacemaker may be a proper choice.^14^ The present case needed high percentage of ventricular pacing and was not infection related; we thus chose to implant a new intravenous electrode aiming at LBBAP. Moreover, this type of lead Medtronic 3830 is much softer and more flexible than the usual ventricular leads and therefore interferes less with tricuspid mobility. Echocardiography 2 days after the lead extraction showed significantly reduced TR accompanied with improvement of symptom.

The 2017 HRS and 2018 EHRA expert consensus statement categorizes indications for CIED-lead extraction into infectious and non-infectious.^15,16^ The non-infectious indication for lead extraction is still controversial considering high risk of complications due to the calcification and fibrosis formation in the lead tips.^17^ However, several case reports and small studies have shown the positive prognosis of CIED-lead extraction in the treatment of severe TR.^8^ Medication alone is unlikely to resolve the diseased conditions of severe TR unless lead-associated mechanical obstruction or valve impingement in the CIED-lead is removed. Our case reinforces the fact that lead extraction is a feasible modality in the treatment of severe TR.

## Conclusion

This case highlights the importance of examining and evaluating the CIED-induced TR. Lead extraction can be a feasible way to treat severe TR caused by tricuspid mechanically lead interference.

## Lead author biography


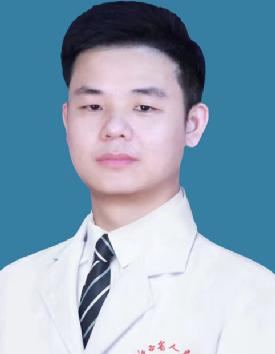


YuLiang Chai, Department of Cardiology, Jiangxi Provincial People’s Hospital, the First Affiliated Hospital of Nanchang Medical College.

**Consent:** The authors confirm that written consent for submission and publication of this report, including the images and associated text, was obtained from the patient in accordance with the COPE guidelines.

**Funding:** None declared.

## Data Availability

The data underlying this report will be shared on reasonable request to the corresponding author.
